# Body Surface Potential Mapping: A Perspective on High‐Density Cutaneous Electrophysiology

**DOI:** 10.1002/advs.202411087

**Published:** 2024-12-16

**Authors:** Ruben Ruiz‐Mateos Serrano, Dario Farina, George G. Malliaras

**Affiliations:** ^1^ Electrical Engineering Division, Department of Engineering University of Cambridge Cambridge CB3 0FA UK; ^2^ Department of Bioengineering Faculty of Engineering, Imperial College London London W12 7TA UK

**Keywords:** BSPM, electrophysiology, high‐density, mapping, wearables

## Abstract

The electrophysiological signals recorded by cutaneous electrodes, known as body surface potentials (BSPs), are widely employed biomarkers in medical diagnosis. Despite their widespread application and success in detecting various conditions, the poor spatial resolution of traditional BSP measurements poses a limit to their diagnostic potential. Advancements in the field of bioelectronics have facilitated the creation of compact, high‐quality, high‐density recording arrays for cutaneous electrophysiology, allowing detailed spatial information acquisition as BSP maps (BSPMs). Currently, the design of electrode arrays for BSP mapping lacks a standardized framework, leading to customizations for each clinical study, limiting comparability, reproducibility, and transferability. This perspective proposes preliminary design guidelines, drawn from existing literature, rooted solely in the physical properties of electrophysiological signals and mathematical principles of signal processing. These guidelines aim to simplify and generalize the optimization process for electrode array design, fostering more effective and applicable clinical research. Moreover, the increased spatial information obtained from BSPMs introduces interpretation challenges. To mitigate this, two strategies are outlined: observational transformations that reconstruct signal sources for intuitive comprehension, and machine learning‐driven diagnostics. BSP mapping offers significant advantages in cutaneous electrophysiology with respect to classic electrophysiological recordings and is expected to expand into broader clinical domains in the future.

## Graphical abstract

This article explores advancements in body surface potential mapping (BSPM), focusing on high‐density electrode arrays. It outlines guidelines for optimal array design based on signal properties and processing principles, addressing spatial resolution and clinical applicability. Challenges in data interpretation are discussed, proposing observational and machine learning approaches for enhanced diagnostic accuracy. The potential of BSPM in transforming cutaneous electrophysiology and expanding its clinical scope is highlighted.

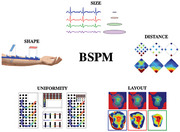

## 1 Main

**Figure 1:**
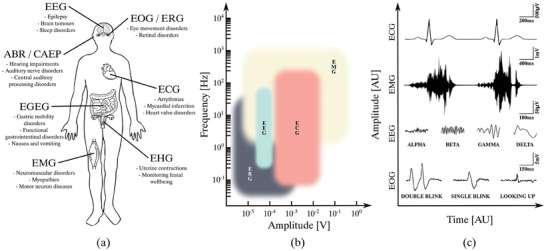
a) Diagram of different body surface potentials in the human body and the conditions they help diagnose. b) Amplitude and spectral characteristics of common body surface potentials, c) Time domain representation of several body surface potentials. EEG = electroencephalography, EOG = electrooculography, ERG = electroretinography, ABR = auditory brainstem responses, CAEP = cortical auditory evoked potentials, ECG = electrocardiography, EGEG = electrogastroenterography, EHG = electrohysterography and EMG = electromyography.

Complex multicellular organisms have developed two main methods of communication to achieve cohesion and synchronicity: chemical, by means of molecular messengers known as hormones; and electrical, by transmitting electrical activity across the membrane of electrically excitable cells (EECs).^[^ ^1^, ^2^, ^3^ ^]^ These are a special group of cells capable of generating electric fields across their bilipid membranes due to controlled imbalances in ionic concentrations inside and outside the cell medium.^[^ ^4^, ^5^ ^]^ The combined electrical activity of EECs propagates outwardly across tissue and can be captured by surface electrodes placed over the skin, yielding a set of time‐varying voltage signals known as body surface potentials (BSPs).^[^ ^6^, ^7^ ^]^ The recording of electrophysiological activity from cutaneous electrodes for clinical purposes has been performed for more than a century and is an essential part of medical diagnosis. Each of these BSPs is obtained at different locations in the body, typically measured relative to a reference electrode away from the source of the investigated signal. As depicted in **Figure** **1**b,c, each BSP pertains to different structures and has different amplitudes, temporal variations, and spectral characteristics.^[^ ^8^, ^9^, ^10^ ^]^

As Figure 1a depicts, there exist multiple types of BSPs employed to diagnose a wide range of conditions. A non‐exhaustive list includes muscle activity from electromyography (EMG), cardiac potentials from electrocardiography (ECG), brain activity from electroencephalography (EEG), gut activity from electrogastroenterography (EGEG), fetal movements from electrohysterography (EHG), eye movements from electrooculography (EOG) and electroretinography (ERG), and hearing activity from auditory brainstem responses (ABRs) and cortical auditory evoked potentials (CAEPs).^[^ ^11^, ^12^ ^]^ As Figure 1b,c depict, each BSP pertains to different structures and has distinct amplitude, temporal variations, and spectral characteristics.

The clinical analysis of BSPs is typically conducted through direct or computer‐aided visual inspection of potentials from individual electrode channels. Computer software, such as LabChart (ADInstruments), MATLAB (MathWorks), LabVIEW (National Instruments), or Python libraries such as “OpenEphys” or “NeuroKit”, are commonly employed to perform the necessary signal processing on BSP signals and display them in a visually intuitive manner. Clinicians learn the expected characteristics of these signals and can identify specific deviations that relate to different pathological conditions (see Figure 1c). Moreover, often a time‐frequency analysis, such as the spectrogram, is performed to investigate the spectral features of the recorded BSPs.^[^ ^13^, ^14^ ^]^ The diagnosis of conditions with BSPs is simple, reproducible, robust, and well‐established across the medical community. However, research in several fields has demonstrated that some of these methods lack sufficient spatial resolution to reliably capture clinically relevant information. For example, conventional 12‐lead ECG is not able to detect pre‐excitation syndromes, late potentials, acute myocardial ischemia, and various electrically isolated wall potential propagation conditions.^[^ ^15^, ^16^, ^17^, ^18^, ^19^, ^20^, ^21^ ^]^ Likewise, bipolar surface EMG is not sufficient to identify individual motor unit activity, fasciculation, and fibrillation potentials,^[^ ^22^, ^23^, ^24^, ^25^ ^]^ and EEG cannot make a fine distinction between different populations of neurons within a particular brain region.^[^ ^26^, ^27^, ^28^ ^]^

**Figure 2:**
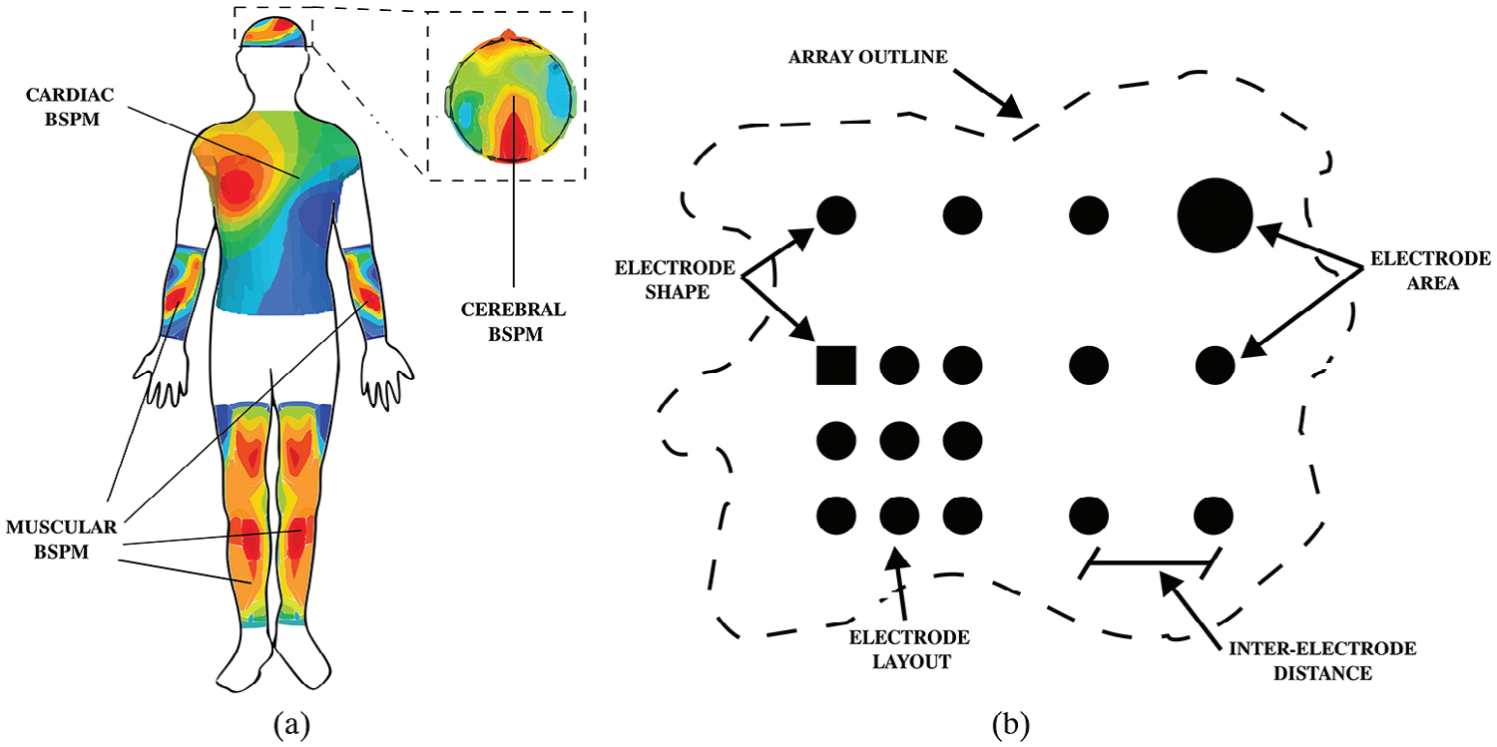
a) Diagram of different body surface potential maps across the human body, along with a sample body surface potential observed by a channel in the map. b) Graphical representation of geometrical electrode array design parameters: array outline, electrode layout, electrode shape, electrode area, and inter‐electrode distance.

Advances in flexible electrode array fabrication techniques and electrode coating materials have enabled the production of conformable, compact, and high‐density arrays for cutaneous electrophysiology, with recording quality exceeding that of state‐of‐the‐art Ag/AgCl electrodes.^[^ ^29^, ^30^, ^31^, ^32^, ^33^, ^34^ ^]^ High signal quality is achieved by minimizing electrode impedance, which directly improves the signal‐to‐noise ratio (SNR) and allows for smaller electrode sizes. Impedance can be reduced by increasing the ionic and electronic conductivity of individual electrodes. Additionally, enhancing the mechanical interface between electrode coatings and the skin reduces impedance and improves signal quality by minimizing motion artifacts.^[^ ^35^, ^36^, ^37^ ^]^ Moreover, these novel fabrication techniques enable robust electromechanical bonding between flexible arrays and rigid external electronics, further enhancing signal quality by minimizing data loss or corruption prior to digitization.^[^ ^38^ ^]^ The BSPs obtained from these arrays can be employed to capture not only temporal and frequency characteristics but also spatial information in the form of detailed BSP heat maps (see **Figure** **2**a). The analysis of these offers a wide range of additional features, such as propagation speeds, channel correlations, or trajectories, which improve diagnostic range and reliability.^[^ ^16^, ^18^, ^19^, ^20^, ^21^, ^23^, ^24^, ^26^, ^27^, ^30^, ^39^, ^40^, ^41^, ^42^, ^43^, ^44^, ^45^, ^46^, ^47^, ^48^, ^49^, ^50^, ^51^, ^52^, ^53^, ^54^, ^55^, ^56^, ^57^, ^58^, ^59^, ^60^, ^61^, ^62^, ^63^, ^64^, ^65^, ^66^, ^67^, ^68^, ^69^, ^70^ ^]^ The independent development of this approach in the ECG, EMG, and EEG fields—showcased through cardiac potential maps, monopolar surface EMG heatmaps, and EEG scalp maps, respectively—reflects the increasing interest within the clinical community to augment traditional diagnostic capabilities of BSP recordings via body surface potential maps (BSPMs)^1^.

The design of electrode arrays for BSP mapping involves the selection of specific parameters, as outlined in Figure 2b. These parameters comprise 1) electrode shape, 2) electrode area, 3) inter‐electrode distance (IED), 4) electrode layout (i.e., maintaining consistent density, area, or shape across an array), and 5) array outline (i.e., determining the body area the electrode array should cover).^[^ ^30^, ^71^, ^72^, ^73^ ^]^ Electrode array design for BSP mapping currently lacks a standardized framework; instead, it is zcustomized for each application and study, where the array parameters are chosen through an experimental iterative process tailored to each specific condition and even each patient.^[^ ^74^, ^75^, ^76^ ^]^ This approach requires an optimization process for every study and renders designs that are useful solely toward the research objectives of the study they were developed for, limiting their comparability, reproducibility, and transferability. A set of electrode array design guidelines for BSP mapping is required to allow for the fast prototyping of devices with optimal geometrical parameters. This general protocol should be agnostic to variability between subjects, body areas, signal range, magnitude, and bandwidth.

This required level of abstraction can only be achieved by defining design rules that are solely based on the physical characteristics of the signals to be recorded. The expected direction of propagation, speed of propagation, and bandwidth of a BSP can be employed to determine the optimal geometrical parameters required for a given array.^[^ ^77^ ^]^ Guidelines implemented under this premise do not present instructions for the design of arrays applicable to any subject or study in any field. Instead, they offer a theoretical framework derived from the underlying properties of the signals under study, providing a preliminary understanding of how to achieve optimal recording of specific electrophysiology signals under given clinical conditions. The objective of this framework is to eliminate the need to empirically engineer arrays for each case study.

**Figure 3:**
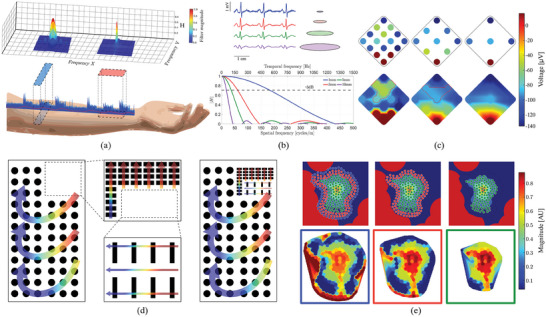
a) Effect of electrode shape on spatial resolution. Top: transfer function for each electrode shape as a function of spatial frequency, bottom: electrode shape projections along with EMG waveforms captured from each. b) Effect of electrode area on spatial resolution and signal‐to‐noise ratio of conventional ECG data. As the area increases the spatial resolution and signal noise decrease due to averaging. The optimal electrode area is the largest area that does not cause an attenuation of −3 dB across the entire signal bandwidth, c) the effect of inter‐electrode distance on spatial resolution. As the density of electrodes decreases, spatial features are not captured, d) Graphical representation of the proposed strategy for the implementation of non‐uniform electrode distribution arrays. The area under study should be subdivided into regions with constant signal physical properties to determine optimal electrode parameters, e) truncation effect, and external signal interference effects caused by spatial under‐ and over‐sampling. Blue: Over‐sampling of the region of interest causes external interference sources to affect recordings, green: under‐sampling causes reconstruction distortions due to lack of information at the boundary, red: a compromise between both effects is achieved by padding the area of interest with a thin additional layer of electrodes.

The proposed guidelines systematically address each of the five geometrical design parameters in electrode arrays (refer to Figure 2b). Electrode shape (1) influences the effective area of individual electrodes, thereby impacting signal attenuation, signal‐to‐noise ratio (SNR), and spatial resolution.^[^ ^30^, ^73^ ^]^ Additionally, it enables the implementation of electrodes with irregular structures or varying axes of symmetry, potentially enhancing overall device conformability and stretchability or reducing electrode insertion complexity (particularly advantageous in implantable applications). While an infinite number of potential geometries can be generated, standard shapes such as circles or rectangles are commonly employed.^[^ ^29^, ^30^, ^33^, ^78^, ^79^, ^80^, ^81^ ^]^ The choice of electrode shape is heavily dependent on the direction of propagation of the signal of interest. As depicted in **Figure** **3**a, signal attenuation in space due to signal averaging within an electrode's covered surface is solely influenced by electrode geometry. The electrode's shape dictates the covered area, thus determining which BSPs are spatially averaged. In scenarios where the direction of signal propagation is known, such as surface EMG in the forearm, shapes can be designed to minimize signal averaging in the direction of propagation (which also minimizes averaging in the time domain) while maximizing electrode area (see Figure 3a). This approach will decrease electrode impedance, improve SNR, and maximize spatial resolution. Conversely, when the signal propagation direction is variable or unknown, a radially symmetric electrode shape (i.e., circular) is preferable, ensuring uniform signal attenuation across all directions (isotropic electrode shape).

The attenuation effect caused by any given electrode geometry on a BSP can be defined quantitatively.^[^ ^82^ ^]^ First, it is necessary to compute the total area ∯ of the electrode, which determines the weight of the averaging operation. The attenuating effect of the weight is then restricted to all locations within the boundary of the electrode (*S_E_*) by defining a piecewise function (*h_E_*), which represents the impulse response of the electrode and encompasses its behaviour against isolated electrical events,

(1) $$h_{E}\left(x,y\right)=\left\{\begin{array}{c} 1/∯S\left(x,y\right)\;dxdy,\;\;x,y\;\in S_{E}\\ 0,\;\;\;\;\;\;\;\;\;\;\;\;\;\;\;\;\;\;\;\;\;\;\;\;\;\;\;\;\;\;\;\;\;\;\;\mathrm{otherwise} \end{array}\right.$$

where *S* represents electrode coverage as a function of position. Equation (1) assumes that the potential distribution under the electrode area is integrated by the electrode, which is valid as a first approximation of the effect of an electrode with physical dimensions and corresponds to a low‐pass filter in the spatial frequency domain. The exact description of electrodes with physical dimensions would imply the solution of a mixed boundary condition problem, but the approximation of pure averaging is sufficient for practical considerations. In order to quantify the frequency dependency of the electrode attenuation on the recorded signal, the 2D Fourier transform can be applied to the impulse response of the electrode to yield its transfer function, $H_{E}\left(f_{x},f_{y}\right)=F\left(h_{E}\left(x,y\right)\right)$, where *f_x_* and *f_y_* are the spatial Fourier frequencies. Examples of transfer functions for circular electrodes of different radii are presented at the bottom of Figure 3b. The case of specific electrode derivations based on linear combinations of signals recorded at different electrodes (e.g., bipolar or Laplacian derivations), can also be treated in the spatial frequency domain (*f_x_*,*f_y_*).^[^ ^83^, ^84^, ^85^ ^]^ If the linear summation of signals detected by different point electrodes is considered, the expression for a spatial filter can be obtained, whose transfer function *H_sf_* (*f_x_*,*f_y_*) is given by:

(2) $$H_{sf}\left(f_{x},f_{y}\right)=\sum_{i=-l}^{q-1}\sum_{u=-g}^{h-1}a_{iu}e^{-jk_{z}id_{x}}\;e^{-jk_{\theta }ud_{y}}$$

with *l*, *q*, *h*, *g* positive integers (*l+q* is the number of electrodes in the *x* direction and *h+g* the number of electrodes in the *y* direction), *a_iu_* the weights given to the electrodes, *d_x_* and *d_y_* the inter‐electrode distances in the two directions (here assumed constant but can be generalized to account for the case where *d_x_* and *d_y_* vary along the grid in order to describe any distribution of electrodes, as shown in Figure 2b). Combining Equations (1) and (2), the effect of electrode shape and spatial filtering can be described by the following transfer function:

(3) $$H_{ele}\left(f_{x},f_{y}\right)=H_{size}\;\left(f_{x},f_{y}\right)H_{sf}\left(f_{x},f_{y}\right)$$

Thus, when a specific derivation (e.g., Laplacian) is applied to specific electrode shapes, the BSP Fourier transform Φ(*f_x_*,*f_y_*) is observed as its spatially filtered version:

(4) $$\begin{array}{ccc} \Phi_{obs}\;\left(f_{x},f_{y}\right) & = & \Phi \left(f_{x},f_{y}\right)H_{ele}\left(f_{x},f_{y}\right)\\ & = & \Phi \left(f_{x},f_{y}\right)H_{size}\left(f_{x},f_{y}\right)H_{sf}\left(f_{x},f_{y}\right) \end{array}$$

Similarly to electrode shape, electrode area (2) significantly impacts both the achievable SNR and spatial resolution. When keeping the shape constant, increasing the electrode area lowers impedance, which enhances SNR. However, this improvement compromises spatial resolution since a larger surface covers more space and reduces the spatial (and therefore temporal) bandwidth since the equivalent low‐pass filter in Equation (1) is more selective for larger areas.^[^ ^30^, ^73^ ^]^ As Figure 3b shows, when a cardiac potential is recorded by electrodes of increasing size, the SNR of the signal improves. However, when the electrode area covers sufficient space to capture multiple relevant signal changes simultaneously, these variations are lost through averaging. To retain essential signal features, the electrode area should remain below a critical threshold. The cut‐off frequency of the low‐pass filter corresponding to the averaging operation should be greater than the spatial bandwidth for each spatial direction. This corresponds to finding an optimal compromise that involves the selection of the largest electrode size that prevents any attenuation of signal features below a −3 dB point within the signal's bandwidth (see Figure 3b). Selecting an area meeting this criteria ensures the highest achievable SNR under the given recording conditions.

The distance between electrodes (3) defines the sensitivity of the array to voltage changes in space, as depicted in Figure 3c, and corresponds to sampling in the spatial domain. Decreasing the IED increases the number of recording points within a given area, enhancing the resolution of local changes in smaller sections.^[^ ^86^ ^]^ When transitioning from BSPs to BSPMs, sampling rate becomes relevant not only in time—measured by the number of data samples obtained within a time interval by recording electronics—but also in space. In theory, reducing the IED will invariably enhance resolution, however, in practice, it will also introduce complexities in electrode wiring, device weight, cost, etc. The optimal solution to this trade‐off is to find the maximum IED (minimum electrode density) that ensures full recoverability of spatial features in the recorded signals. The Nyquist‐Shannon theorem, extensively utilized for determining the minimum temporal sampling rate required for perfect signal reconstruction, dictates that signals sampled at more than twice the highest frequency produced by the signal of interest are entirely recoverable.^[^ ^87^ ^]^ The frequency bandwidth in the time domain of most physiological signals has been characterized, so the maximum frequency of each BSP can be determined. The question that arises is whether a relationship between temporal and spatial sampling rates can be established to ascertain the most suitable IED for BSP recording. Under the assumption of constant BSP propagation speeds, it is possible to relate time and space by means of the conduction velocity (CV) of the propagating potential. The CV is the speed at which signals travel through tissue. For BSPs, CV varies between ≈3 and 5 m s^−1^ for most signals.^[^ ^73^, ^88^, ^89^ ^]^ Slower BSP propagation speeds cause changes in potential across time to become less sparse in space, necessitating a greater number of electrodes to detect them. Assuming the lowest physiologically feasible CV is recommended to prevent aliasing effects at lower CV values. Establishing a relationship between temporal sampling frequency (*f*) and IED through the CV can be achieved by means of a simple proportion, IED = CV/*f*. This association is valid for the spatial direction of propagation of the BSP but not in the other spatial direction.

The threshold IED value obtained from this calculation does not account for practical scenarios where the IED may not be maintained perfectly across all electrodes in the array. Deviations from the threshold can result in distances slightly exceeding or falling short of the minimum required for optimal interpolation. When the distance is below the threshold, interpolation will be accurate; however, when the distance exceeds the threshold, interpolation will be imperfect, leading to reconstruction errors proportional to the deviation in the electrode position. If the expected deviation in electrode positioning is known, it is advisable to reduce the IED by the deviation value to avoid interpolation errors.

The optimization of electrode layout (4) facilitates the creation of devices featuring distinct sections, each tailored to capture signals exhibiting varying physical properties. The choice of this parameter is primarily dependent on the behaviour exhibited by the signal of interest across the recording area. Adjusting electrode layout can potentially impact electrode shape, area, and IED. The previously outlined guidelines for these parameters can be employed as a framework to evaluate the necessity for greater or lesser electrode layout. When considering this parameter, it is recommended to start by examining the entire area of interest, investigating whether the signal properties—direction of propagation, bandwidth, and CV—can be assumed to remain consistent or vary predictably. If only the direction of propagation varies, it is relevant to assess whether the variability is unknown or consistent in the form of a known trajectory. In the former case, a uniform array with circular‐shaped electrodes might be preferable; in the latter, a non‐uniform variation in electrode shape might be required. When two or more properties exhibit well‐characterized variations, a proposed methodology involves segmenting the total area of interest into smaller sections. Each section undergoes a similar analytical process as described above, leading to either a defined set of design values (if the three properties can be assumed to remain constant) or a further breakdown of the section. This iterative process continues until design values are established for all sections within the array, see a graphical representation in Figure 3d.

The final design choice, array outline (5), determines the body area the electrode array should cover. This differs from the outline of the entire device, which must be adaptable to accommodate diverse individuals and clinical setups. Square lattices are commonly used in the literature because they simplify the process of importing the data into the software, as each row and column in a matrix can directly represent a specific location in space.^[^ ^29^, ^30^, ^33^, ^78^, ^79^, ^81^ ^]^ However, it is advisable to employ hexagonal or triangular lattices as they maintain a consistent IED between neighbouring electrodes (see Figure 3c for an example of a square lattice, where the diagonal distance is greater than the lateral one). The selection of an array outline is ultimately dependent on the proximity of interferent anatomical structures and the spatial sampling rate of the array. Limiting the coverage of the array to the area of interest alone leads to distortion or aliasing effects (since the spatial bandwidth becomes infinite in theory) at the edges of recorded BSPMs, a phenomenon known as truncation.^[^ ^73^ ^]^ This reconstruction effect occurs due to a lack of information about the area surrounding boundary electrodes, which causes estimations of the potential values around the edges to be inaccurate. Conversely, excessively broad coverage of the surrounding area will capture unwanted signals from external sources, potentially impacting diagnosis or subsequent processing. Figure 3e illustrates examples of distortions caused by both extremes. In order to mitigate both truncation and interference, it is recommended to extend the spatial sampling of the array to include at least one additional point around the edge of the area of interest.

The aforementioned guidelines provide a set of preliminary design choices based exclusively on the physical properties of electrophysiological signals and mathematical principles governing signal processing. Their aim is to simplify and generalize the optimization process for electrode array design to allow for more effective and translatable clinical research. The optimization of electrode array parameters remains necessary since the underlying assumptions on which they are based require experimental validation.

As highlighted earlier, there is a clear tendency in the clinical community to employ BSPM over traditional BSPs since augmented spatial information improves diagnostic range and precision.^[^ ^16^, ^18^, ^19^, ^20^, ^21^, ^23^, ^24^, ^26^, ^27^, ^30^, ^39^, ^40^, ^41^, ^42^, ^43^, ^44^, ^45^, ^46^, ^47^, ^48^, ^49^, ^50^, ^51^, ^52^, ^53^, ^54^, ^55^, ^56^, ^57^, ^58^, ^59^, ^60^, ^61^, ^62^, ^63^, ^64^, ^65^, ^66^, ^67^, ^68^, ^69^, ^70^ ^]^ However, this transition comes with challenges, including the management of increased cable wiring density and connectivity.^[^ ^80^, ^90^, ^91^ ^]^ Wireless data transmission has been widely explored in the literature as a solution to these connectivity issues, enhancing the wearability and comfort of non‐invasive devices.^[^ ^92^, ^93^ ^]^ A wide range of communication protocols, hardware, firmware, and software tools are available for implementing wireless systems. Additionally, flexible electronic fabrication techniques have been adapted to enable integrated on‐device wireless systems.^[^ ^94^, ^95^ ^]^ However, wireless data transmission faces data rate limitations as the number of simultaneous recording channels increases. While the low‐frequency band of cutaneous electrophysiological signals allows for a relatively low sampling rate that can support tens of channels, it may not suffice for arrays with hundreds or thousands of channels, restricting its use in several BSP mapping applications.^[^ ^96^ ^]^

The spatial nature of BSPMs allows intuitive observation of activity recorded by hundreds of channels since areas with different activity display varying intensity levels. Nevertheless, the interpretation of these potential values for diagnostic purposes results challenging. On the one hand, there is no precedent for what a healthy BSPM reference should resemble, the potentials measured at the surface of the skin do not immediately represent independent EECs but are a combination (linear or non‐linear, depending on the type of potentials) of the activity of multiple sources propagated through tissue.^[^ ^97^, ^98^, ^99^ ^]^ On the other hand, there is no prior knowledge of what biomarkers or BSPM features are relevant to identifying different pathological conditions.^[^ ^100^ ^]^ It becomes the task of specialized clinical field experts to identify this information by means of multiple case studies and spread it to a wider scientific community. This process is slow and renders the devices impractical in the short‐term.

**Figure 4:**
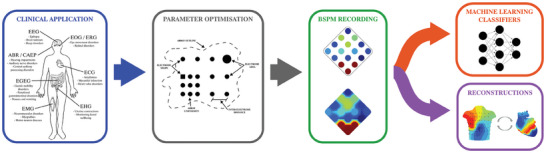
The process of BSP mapping in clinical diagnosis. First, a clinical application is targeted. Second, the geometrical parameters of the BSPM arrays are optimized for the given application. Third, the BSPs are recorded and BSPMs are generated through interpolation. Once the data is gathered two strategies for data interpretation can be taken: a diagnostic approach, in which BSPM data is employed directly to train machine learning classifiers able to diagnose conditions, and an observational approach, in which BSPM data is transformed to simplify human diagnosis.

As **Figure** **4** illustrates, two approaches can be employed to overcome the interpretability barrier in BSPM. The first is observational and focuses on modifying how BSPM data is visualized to enable a more intuitive understanding of potential values. This can be achieved through transformations or reconstructions that clarify the independent sources generating the data. For example, surface EMG monopolar array data can be transformed to yield independent motor unit activity, and cardiac BSPMs can be reconstructed using ECG imaging to visualize epicardial potentials.^[^ ^22^, ^23^, ^62^, ^66^, ^101^, ^102^, ^103^ ^]^ This approach allows for direct human inspection and the discovery of unknown conditions; however, it requires the development of transformation and reconstruction algorithms to solve the inverse problem of electrophysiology. This problem is mathematically ill‐posed, making it challenging to find biophysically plausible and accurate solutions.^[^ ^97^, ^98^, ^99^, ^104^ ^]^

The second method is diagnostic, leveraging machine learning (ML) algorithms that use BSPM data directly or extracted features to autonomously classify known conditions or determine their progression.^[^ ^105^ ^]^ Training paradigms for classical ML classifiers fall into three categories: supervised learning, unsupervised learning, and reinforcement learning.^[^ ^106^ ^]^ Supervised learning uses labelled input data to define the relationship between an input and its corresponding class, with algorithms including linear and logistic regression, support vector machines, decision trees, random forests, artificial neural networks, naïve Bayes classifiers, AdaBoost, and ensemble methods.^[^ ^107^ ^]^ Unsupervised learning employs statistical methods, such as clustering or dimensionality reduction, to define distinct classes from unlabelled data, with examples including K‐means, principal component analysis (PCA), and singular value decomposition (SVD).^[^ ^108^ ^]^ Reinforcement learning involves an agent exploring an unknown parameter space to determine the optimal policy that maximizes reward, continuously improving through experience. Examples include Markov decision processes, Q‐learning, and Monte Carlo methods.^[^ ^109^ ^]^

Classical machine learning methods aim to establish relationships between known correlated inputs and outputs, relying on predefined features or biomarkers relevant to the condition being classified. As previously mentioned, in traditional BSP analysis clinicians have empirically identified these features, allowing for visual diagnosis. However, in the context of BSPM, there is often little prior knowledge about which features are crucial for diagnosis, making many classical ML methods unsuitable for direct application. While non‐deep learning approaches can be useful where domain‐specific knowledge allows for effective feature engineering, their applicability is limited in the absence of clear biomarkers.^[^ ^110^ ^]^ In contrast, deep learning methods excel in such scenarios, as they can automatically extract complex features from raw BSPM data without prior knowledge.^[^ ^111^ ^]^ With their multi‐layered architecture, these networks identify patterns and establish relationships between the data and diagnostic outcomes in ways that traditional methods cannot match.

The implementation of deep learning methods in clinical environments presents significant challenges. One major issue is the requirement for large, labelled datasets and substantial computational resources, both of which are often limited in healthcare settings where data collection can be fragmented or inconsistent.^[^ ^112^ ^]^ Additionally, working with large‐scale data raises critical concerns about patient privacy. Various approaches have been developed to address these concerns. Data anonymization, where personally identifiable information (PII) is removed or encrypted, is commonly used; however, anonymized data can sometimes be re‐identified through sophisticated inference techniques.^[^ ^113^, ^114^ ^]^ To mitigate this risk, differential privacy can be employed, adding controlled noise to the data to protect individual identities while maintaining the accuracy of aggregate trends.^[^ ^115^ ^]^ Another promising technique is federated learning, which allows models to be trained locally on patient devices or within hospital systems, ensuring that sensitive data never leaves its source by sharing only model updates with a central server.^[^ ^116^ ^]^ Homomorphic encryption also enables secure computations on encrypted data without revealing raw information.^[^ ^117^, ^118^ ^]^ These privacy‐preserving methods are crucial to ensuring that large‐scale data can be used for research and diagnostics without compromising patient confidentiality.

Beyond data requirements, another key challenge for deep learning is its “black‐box” nature, which limits interpretability. While these models can achieve high diagnostic accuracy, clinicians often struggle to understand which features in the data drive predictions, leading to a lack of trust in AI‐based decisions.^[^ ^119^ ^]^ This challenge has spurred growing interest in explainable AI (XAI), which aims to make deep learning models more transparent.^[^ ^120^ ^]^ Techniques such as saliency maps, feature attribution, and attention mechanisms have been proposed to help clinicians identify relevant features in complex clinical data.^[^ ^121^, ^122^, ^123^, ^124^, ^125^ ^]^ Additionally, integrating deep learning into clinical workflows requires addressing practical challenges, including real‐time data processing, creating user‐friendly interfaces, and validating models across diverse patient populations.^[^ ^126^, ^127^, ^128^ ^]^

The most promising direction for the future may lie in developing hybrid models that combine the strengths of deep learning and traditional methods.^[^ ^129^, ^130^, ^131^, ^132^, ^133^ ^]^ These hybrid approaches could harness deep learning's capacity to extract complex features from BSPM data while integrating more interpretable techniques, such as classical machine learning or rule‐based systems, to enhance transparency and clinical trust. By striking a balance between accuracy and interpretability, hybrid models can bridge the gap between cutting‐edge computational power and practical clinical application.

As has been described, both observational and diagnostic methods in BSPM present distinct advantages and drawbacks that impact their clinical utility. Observational models enhance diagnostic insight by offering greater transparency and enabling clinicians to visualize electrophysiological data through transformations and reconstructions. This approach is particularly beneficial for diagnosing complex conditions, as it allows for the identification of nuanced patterns that might be overlooked in raw data. However, these models face challenges due to the mathematical complexity of solving inverse problems, which can lead to inaccurate reconstructions. Therefore, future developments must enhance the accuracy and stability of these transformations while integrating intuitive visualization tools into clinical workflows, allowing for seamless interpretation without requiring extensive computational expertise.

Conversely, the diagnostic approach, driven by machine learning, can significantly improve efficiency and accuracy by rapidly processing large volumes of BSPM data to identify patterns that may not be immediately apparent to human observers. This capability is especially crucial for conditions with subtle or early‐stage manifestations, where early detection can enhance patient outcomes. As BSPM technology advances and data volume increases due to high‐density arrays, the robustness of diagnostic algorithms becomes even more valuable. Future efforts will need to ensure high diagnostic accuracy while also addressing interpretability concerns to foster clinician trust in automated systems. Additionally, optimizing the integration of ML models into clinical workflows will be essential for ensuring these tools complement traditional diagnostic methods and enhance overall clinical practice. Ultimately, the ongoing development of both approaches will create unique opportunities to improve clinical diagnosis, each addressing specific implications and challenges in the evolving landscape of BSPM technology.

With future advancements in data acquisition and sensor technology, both the observational and diagnostic approaches in BSPM are set to improve significantly. As BSPM arrays become more compact, flexible, and capable of capturing higher‐density signals, the volume and quality of data will improve for both methods. This enhancement will boost the diagnostic accuracy of ML models and provide clinicians with more detailed visualizations through the observational approach. While the observational method facilitates discovering unknown conditions and generating hypotheses, the diagnostic approach excels in automating decision‐making for known conditions. The future of BSPM in clinical diagnosis will likely see a synergistic integration of both strategies, where visualization techniques can reveal novel patterns in the data that ML models can analyze, and classify. Conversely, automated diagnostic systems could identify potential anomalies, prompting clinicians to investigate further using observational tools. By merging improved spatial information with advanced computational methods, BSPM will enable earlier detection of pathologies, more accurate staging of disease progression, and personalized treatment planning. The continued development of both observational and diagnostic approaches will be essential to realizing this potential, solidifying BSPM as a cornerstone of future clinical diagnostics.

## 2 Conclusion

The recording of BSPs from cutaneous electrodes stands as an essential part of medical diagnosis. Despite its widespread use and success in treating numerous diseases, traditional BSPs have been shown to lack sufficient spatial resolution to capture several conditions. This limitation has produced the simultaneous, independent development of electrode array recording techniques across multiple clinical fields where spatial information is acquired in the form of BSPMs. Presently, the design of electrode arrays for BSP mapping lacks a standardized framework, resulting in customizations for each clinical study, thereby limiting comparability, reproducibility, and transferability. In this study, a set of preliminary design guidelines, derived from existing literature, have been proposed. These rules are based exclusively on the physical properties of electrophysiological signals and the mathematical principles of signal processing. Their purpose is to simplify and generalize the optimization process for electrode array design, enabling more effective and translatable clinical research. The increased spatial information obtained through BSPMs introduces challenges in interpretation. To address this, two strategies have been outlined: observational transformations that reconstruct signal sources for intuitive comprehension and machine learning‐driven diagnostics for condition discernment. Each strategy presents distinct advantages and drawbacks, selection of each should be determined by precise clinical objectives. BSP mapping presents significant advantages in cutaneous electrophysiology and is anticipated to expand into broader clinical domains in the forthcoming decades.

## Conflict of Interest

The authors declare no conflict of interest.

## References

[1] E. R. Kandel , J. H. Schwartz , T. M. Jessell , S. A. Siegelbaum , A. J. Hudspeth , Principles of Neural Science, McGraw‐Hill, New York, NY, 2013.

[2] B. Alberts , A. Johnson , J. Lewis , M. Raff , K. Roberts , P. Walter , Molecular Biology of the Cell, Garland Science, New York, NY, 2014.

[3] A. C. Guyton , J. E. Hall , Textbook of Medical Physiology, Elsevier, Philadelphia, PA, 2020.

[4] N. Sperelakis , J. C. Freedman , Cell Physiology Source Book, Academic Press, San Diego, CA, 2011.

[5] W. D. Stein , Membrane Physiology, Cambridge University Press, Cambridge, UK, 2012.

[6] J. D. Bronzino , R. C. Barr , B. J. Roth , A. Varghese , C. R. Johnson , W. M. Smith , E. J. Berbari , K.‐Å. Henneberg , The Biomedical Engineering Handbook, CRC Press, Boca Raton, FL, 2014.

[7] F. Lombardi , Electrophysiological Recording Techniques, Springer, New York, NY, 2016.

[8] P. Denes , M. D. Ezri , J Am Coll Cardiol 1983, 1, 292. 6826939 10.1016/s0735-1097(83)80030-8

[9] E. Kaniusas , Biomedical Signals and Sensors I: Linking Physiological Phenomena and Biosignals, Springer, Berlin, Germany, 2012.

[10] G. J. Klein , Clinical Electrophysiology Review, McGraw‐Hill Education, New York, NY, 2016.

[11] R. B. Reilly , T. C. Lee , Technology and Health Care 2010, 18, 443. 21099006 10.3233/THC-2010-0604

[12] N. V. Thakor , Biopotentials and Electrophysiology Measurements, CRC Press, Boca Raton, FL, 2015.

[13] S. Szava , P. Valdes , R. Biscay , L. Galan , J. Bosch , I. Clark , J. C. Jimenez , Brain Topogr 1994, 6, 211. 8204408 10.1007/BF01187711

[14] R. Gao , J Neurophysiol 2016, 115, 628. 26245320 10.1152/jn.00722.2015 PMC4752306

[15] B. Taccardi , Circ. Res. 1963, 12, 341. 13980115 10.1161/01.res.12.4.341

[16] L. S. Green , R. L. Lux , C. W. Haws , Circulation 1987, 76, 1290. 3677354 10.1161/01.cir.76.6.1290

[17] D. Speake , P. Terry , Emergency Medicine Journal 2001, 18, 61. 10.1136/emj.18.1.61-a PMC1725513 11310469

[18] W. J. Brady , T. P. Aufderheide , T. Chan , A. D. Perron , Emerg Med Clin North Am 2001, 19, 295. 11373980 10.1016/s0733-8627(05)70185-1

[19] R. P. Ganim , W. R. Lewis , D. B. Diercks , D. Kirk , R. Sabapathy , L. Baker , E. A. Amsterdam , Cardiology 2004, 102, 100. 15103180 10.1159/000077912

[20] J. P. Ornato , I. B. A. Menown , M. A. Peberdy , M. C. Kontos , J. W. Riddell , G. L. Higgins , S. J. Maynard , J. Adgey , Am J Emerg Med 2009, 27, 779. 19683104 10.1016/j.ajem.2008.06.010

[21] E. Szűcs , K. Szakolczai , G. Simonyi , T. Bauernfeind , A. Pintér , I. Préda , M. Medvegy , J Electrocardiol 2010, 43, 326. 20381065 10.1016/j.jelectrocard.2010.02.002

[22] G. Yue , A. J. Fuglevand , M. A. Nordstrom , R. M. Enoka , Biol. Cybern. 1995, 73, 223. 7548311 10.1007/BF00201424

[23] R. Merletti , A. Holobar , D. Farina , Journal of electromyography and kinesiology 2008, 18, 879. 19004645 10.1016/j.jelekin.2008.09.002

[24] R. Merletti , A. Botter , C. Cescon , M. A. Minetto , T. M. M. Vieira , Crit Rev Biomed Eng 2010, 38, 347. 10.1615/critrevbiomedeng.v38.i4.20 21133838

[25] R. Merletti , D. Farina , Surface Electromyography: Physiology, Engineering, and Applications, John Wiley & Sons, Hoboken, New Jersey, 2016.

[26] D. Lehmann , International Journal of Psychophysiology 1984, 1, 267. 6542911 10.1016/0167-8760(84)90046-1

[27] D. Yao , Z. Yin , X. Tang , L. Arendt‐Nielsen , A. C. N. Chen , Phys Med Biol 2004, 49, 5073. 15609559 10.1088/0031-9155/49/22/004

[28] V. Janiukstyte , T. W. Owen , U. J. Chaudhary , B. Diehl , L. Lemieux , J. S. Duncan , J. de Tisi , Y. Wang , P. N. Taylor , Sci. Rep. 2023, 13, 13442. 37596291 10.1038/s41598-023-39700-7 PMC10439201

[29] S. Velasco‐Bosom , N. Karam , A. Carnicer‐Lombarte , J. Gurke , N. Casado , L. C. Tomé , D. Mecerreyes , G. G. Malliaras , Adv. Healthcare Mater. 2021, 10, 2100374. 10.1002/adhm.202100374 PMC11469138 33991046

[30] R. Ruiz‐Mateos Serrano , S. Velasco‐Bosom , A. Dominguez‐Alfaro , M. L. Picchio , D. Mantione , D. Mecerreyes , G. G. Malliaras , Adv. Sci. 2023, 11, 2301176. 10.1002/advs.202301176 PMC11251564 37203308

[31] J. L. de Lacalle , M. L. Picchio , A. Dominguez‐Alfaro , R. R. M. Serrano , B. Marchiori , I. del Agua , N. Lopez‐Larrea , M. Criado‐Gonzalez , G. G. Malliaras , D. Mecerreyes , ACS Mater. Lett. 2023, 5, 3340. 38075386 10.1021/acsmaterialslett.3c00938 PMC10698741

[32] A. Dominguez‐Alfaro , E. Mitoudi‐Vagourdi , I. Dimov , M. L. Picchio , N. Lopez‐Larrea , J. L. de Lacalle , X. Tao , R. R. M. Serrano , A. Gallastegui , N. Vassardanis , D. Mecerreyes , G. G. Malliaras , Adv. Sci. 2024, 11, 2306424. 10.1002/advs.202306424 PMC11251555 38251224

[33] R. Ruiz‐Mateos Serrano , A. Aguzin , E. Mitoudi‐Vagourdi , X. Tao , T. E. Naegele , A. T. Jin , N. Lopez‐Larrea , M. L. Picchio , M. Vinicio Alban‐Paccha , R. J. Minari , D. Mecerreyes , A. Dominguez‐Alfaro , G. G. Malliaras , Biomaterials 2024, 310, 122624. 38805956 10.1016/j.biomaterials.2024.122624

[34] C. Slaughter , S. Velasco‐Bosom , X. Tao , R. Ruiz‐Mateos Serrano , S. Kissovsky , R. Mizuta , D. Mantione , S. T. Keene , G. G. Malliaras , A. Dominguez‐Alfaro , J Mater Chem C Mater 2024, 12, 14944.

[35] S. Chun , D.a W. Kim , S. Baik , H. J. Lee , J. H. Lee , S. H.o Bhang , C. Pang , Adv. Funct. Mater. 2018, 28, 1805224.

[36] F. Stauffer , M. Thielen , C. Sauter , S. Chardonnens , S. Bachmann , K. Tybrandt , C. Peters , C. Hierold , J. Vörös , Adv. Healthcare Mater. 2018, 7, 1700994. 10.1002/adhm.201700994 29330962

[37] A. Aguzin , A. Dominguez‐Alfaro , M. Criado‐Gonzalez , S. Velasco‐Bosom , M. L. Picchio , N. Casado , E. Mitoudi‐Vagourdi , R. J. Minari , G. G. Malliaras , D. Mecerreyes , Mater. Horiz. 2023, 10, 2516. 37067040 10.1039/d3mh00310h

[38] C. Chitrakar , E. Hedrick , L. Adegoke , M. Ecker , Materials 2022, 15, 1664. 35268893 10.3390/ma15051664 PMC8911085

[39] L. De Ambroggi , B. Taccardi , E. Macchi , Circulation 1976, 54, 251. 181170 10.1161/01.cir.54.2.251

[40] D. M. Mirvis , F. W. Keller , R. E. Ideker , J. W. Cox , D. G. Zettergren , R. F. Dowdie , J Electrocardiol 1977, 10, 347. 915403 10.1016/s0022-0736(77)80008-3

[41] T. J. Montague , J. P. Finley , K. Mukelabai , S. A. Black , S. M. Rigby , C. A. Spencer , B. M. Horacek , Am. J. Cardiol. 1984, 54, 301. 6465009 10.1016/0002-9149(84)90187-5

[42] D. D. McPherson , B. M. Horacek , C. A. Spencer , D. E. Johnstone , L. D. Lalonde , C. L. Cousins , T. J. Montague , Chest 1985, 88, 841. 4064772 10.1378/chest.88.6.841

[43] M. J. Gardner , T. J. Montague , S. Armstrong , B. M. Horacek , E. R. Smith , Circulation 1986, 73, 684. 3948371 10.1161/01.cir.73.4.684

[44] L. de Ambroggi , T. Bertoni , E. Locati , M. Stramba‐Badiale , P. J. Schwartz , Circulation 1986, 74, 1334. 3779919 10.1161/01.cir.74.6.1334

[45] D. D. McPherson , B. M. Horacek , D. E. Johnstone , L. D. Lalonde , C. A. Spencer , T. J. Montague , Can. J. Cardiol. 1986, 8, 521. 10.1016/s0735-1097(86)80178-4 2875088

[46] T. J. Montague , D. E. Johnstone , C. A. Spencer , L. D. Lalonde , M. J. Gardner , M. G. O'Reilly , B. M. Horacek , Am. J. Cardiol. 1986, 58, 1173. 3788804 10.1016/0002-9149(86)90377-2

[47] T. J. Montague , D. E. Johnstone , C. A. Spencer , R. M. Miller , B. R. Mackenzie , M. J. Gardner , B. M. Horacek , Am. J. Cardiol. 1988, 61, 273. 3341203 10.1016/0002-9149(88)90930-7

[48] L. De Ambroggi , T. Bertoni , M. L. Breghi , M. Marconi , M. Mosca , J Electrocardiol 1988, 21, 321. 3241143 10.1016/0022-0736(88)90108-2

[49] T. J. Montague , F. X. Witkowski , Am. J. Cardiol. 1989, 64, 378. 2526994 10.1016/0002-9149(89)90539-0

[50] T. J. Montague , F. X. Witkowski , R. M. Miller , D. E. Johnstone , R. B. MacKenzie , C. A. Spencer , B. M. Horacek , Chest 1990, 97, 1333. 2347218 10.1378/chest.97.6.1333

[51] F. Kornreich , T. J. Montague , P. M. Rautaharju , J Electrocardiol 1992, 25, 15. 1297686 10.1016/0022-0736(92)90051-z

[52] F. Kornreich , T. J. Montague , P. M. Rautaharju , Circulation 1993, 87, 773. 8443898 10.1161/01.cir.87.3.773

[53] R. L. Lux , L. S. Green , R. S. MacLeod , B. Taccardi , J Electrocardiol 1994, 27, 100. 7884342 10.1016/s0022-0736(94)80065-0

[54] C. L. Hubley‐Kozey , L. B. Mitchell , M. J. Gardner , J. W. Warren , C. J. Penney , E. R. Smith , B. M. Horácek , Circulation 1995, 92, 1825. 7671367 10.1161/01.cir.92.7.1825

[55] L. De Ambroggi , E. Aimè , C. Ceriotti , M. Rovida , S. Negroni , Circulation 1997, 96, 4314. 9416898 10.1161/01.cir.96.12.4314

[56] C. Babiloni , F. Carducci , F. Cincotti , P. M. Rossini , C. Neuper , G. Pfurtscheller , F. Babiloni , Neuroimage 1999, 10, 658. 10600411 10.1006/nimg.1999.0504

[57] R. L. Lux , M. S. Fuller , R. S. MacLeod , P. R. Ershler , B. B. Punske , B. Taccardi , J Electrocardiol 1999, 32, 153. 10688319 10.1016/s0022-0736(99)90073-0

[58] C. Babiloni , F. Babiloni , F. Carducci , F. Cincotti , G. Cocozza , C. Del Percio , D. V. Moretti , P. M. Rossini , Neuroimage 2002, 17, 559. 12377134

[59] D. D. Finlay , C. D. Nugent , P. J. McCullagh , N. D. Black , Biomed. Eng. Online 2005, 4, 51. 16138921 10.1186/1475-925X-4-51 PMC1208920

[60] G. Drost , D. F. Stegeman , B. G. M. van Engelen , M. J. Zwarts , J Electromyogr Kinesiol 2006, 16, 586. 17085302 10.1016/j.jelekin.2006.09.005

[61] L. Astolfi , F. Cincotti , D. Mattia , M. G. Marciani , L. A. Baccala , F. de Vico Fallani , S. Salinari , M. Ursino , M. Zavaglia , L. Ding , J. C. Edgar , G. A. Miller , B. He , F. Babiloni , Hum Brain Mapp 2007, 28, 143. 16761264 10.1002/hbm.20263 PMC6871398

[62] E. M. Maathuis , J. Drenthen , J. P. van Dijk , G. H. Visser , J. H. Blok , J Electromyography and Kinesiology 2008, 18, 920. 10.1016/j.jelekin.2008.09.001 18996724

[63] M. D. Holmes , Epilepsia 2008, 49, 3.

[64] M. R. Robinson , N. Curzen , Ann Noninvasive Electrocardiol 2009, 14, 201. 19419406 10.1111/j.1542-474X.2009.00284.x PMC6932397

[65] M. Rojas‐Martinez , M. A. Mañanas , J. F. Alonso , J Neuroeng Rehabil 2012, 9, 85. 23216679 10.1186/1743-0003-9-85 PMC3575258

[66] D. F. Stegeman , B. U. Kleine , B. G. Lapatki , J. P. Van Dijk , Biocybern Biomed Eng 2012, 32, 3.

[67] M. J. Franks , L. Lawson , Adv Emerg Nurs J 2012, 34, 32. 22313899 10.1097/TME.0b013e31823df79a

[68] Z. F. Issa , J. M. Miller , D. P. Zipes , Clinical Arrhythmology and Electrophysiology: A Companion to Braunwald's Heart Disease, 2nd Edition Elsevier, Amsterdam, Netherlands 2012.

[69] A. J. Bank , R. M. Gage , A. E. Curtin , K. V. Burns , J. M. Gillberg , S. Ghosh , J Electrocardiol 2018, 51, 534. 29273234 10.1016/j.jelectrocard.2017.12.004

[70] C. M. Michel , Handb Clin Neurol 2019, 160, 185. 31277847 10.1016/B978-0-444-64032-1.00012-6

[71] J. N. Helal , P. Bouissou , IEEE Trans. Biomed. Eng. 1992, 39, 1161. 1487279 10.1109/10.168695

[72] B. Afsharipour , K. Ullah , R. Merletti , Biomed Signal Process Control 2015, 22, 170.

[73] B. Afsharipour , S. Soedirdjo , R. Merletti , Biomed Signal Process Control 2019, 49, 298.

[74] D. Staudenmann , I. Kingma , D. F. Stegeman , J. H. van Dieën , J. Electromyography and Kinesiolo, gy 2005, 15, 1. 10.1016/j.jelekin.2004.06.008 15642649

[75] A. Andrews , E. Morin , L. McLean , EMBC 2009, 2987. 19963553 10.1109/IEMBS.2009.5332520

[76] M. Besomi , P. W. Hodges , J. Van Dieën , R. G. Carson , E. A. Clancy , C. Disselhorst‐Klug , A. Holobar , F. Hug , M. C. Kiernan , M. Lowery , K. McGill , R. Merletti , E. Perreault , K. Søgaard , K. Tucker , T. Besier , R. Enoka , D. Falla , D. Farina , S. Gandevia , J. C. Rothwell , B. Vicenzino , T. Wrigley , J. Electromyography and Kinesiology 2019, 48, 128. 10.1016/j.jelekin.2019.07.008 31352156

[77] R. Merletti , L. Lo Conte , E. Avignone , P. Guglielminotti , IEEE Trans. Biomed. Eng. 1999, 46, 810. 10396899 10.1109/10.771190

[78] B. G. Lapatki , J. P. Van Dijk , I. E. Jonas , M. J. Zwarts , D. F. Stegeman , J. Appl. Physiol. 2004, 96, 327. 12972436 10.1152/japplphysiol.00521.2003

[79] M. Lee , D. Kim , H. S. Shin , H. G. Sung , J. H. Choi , JoVE (J. Visualized Experiments) 2011, 47, e2562. 10.3791/2562 PMC3182650 21248705

[80] S. Tam , G. Bilodeau , J. Brown , G. Gagnon‐Turcotte , A. Campeau‐Lecours , B. Gosselin , in 41st Annual Int. Conf. of the IEEE Engineering in Medicine and Biology Society (EMBC), IEEE, Berlin, Germany 2019, pp. 6040–6044. 10.1109/EMBC.2019.8857750 31947223

[81] J. E. Lara , L. K. Cheng , O. Röhrle , N. Paskaranandavadivel , IEEE Trans. Biomed. Eng. 2022, 69, 1758. 34847014 10.1109/TBME.2021.3131297

[82] J. N. Helal , P. Bouissou , IEEE Trans. Biomed. Eng. 1992, 39, 1161. 1487279 10.1109/10.168695

[83] D. Farina , R. Merletti , IEEE Trans. Biomed. Eng. 2001, 48, 637. 11396594 10.1109/10.923782

[84] D. Farina , R. Merletti , Acta Physiol Pharmacol Bulg 2001, 26, 63. 11693404

[85] D. Farina , L. Mesin , S. Martina , R. Merletti , IEEE Trans. Biomed. Eng. 2004, 51, 415. 15000373 10.1109/TBME.2003.820998

[86] S. Usui , H. Araki , IEEE Engineering in Medicine and Biology Magazine 1990, 9, 29. 10.1109/51.62900 18238313

[87] C. E. Shannon , Proceedings of the IRE 1949, 37, 10.

[88] N. Sperelakis , L. Ramasamy , Theor Biol Med Model 2006, 3, 29. 16911777 10.1186/1742-4682-3-29 PMC1578564

[89] T. Asakawa , A. Muramatsu , T. Hayashi , T. Urata , M. Taya , Y. Mizuno‐Matsumoto , Front Hum Neurosci 2014, 8, 1006. 25540618 10.3389/fnhum.2014.01006 PMC4261731

[90] U. Barone , R. Merletti , IEEE Trans. Biomed. Eng. 2013, 60, 2242. 23508246 10.1109/TBME.2013.2252346

[91] A. Moin , A. Zhou , A. Rahimi , S. Benatti , A. Menon , S. Tamakloe , J. Ting , N. Yamamoto , Y. Khan , F. Burghardt , et al., in 2018 IEEE Int. Symposium on Circuits and Systems (ISCAS), IEEE, Florence, Italy 2018, pp. 1–5.

[92] Y. G. Park , S. Lee , J. U. Park , Sensors 2019, 19, 4353.31600870

[93] L. Kong , W. Li , T. Zhang , H. Ma , Y. Cao , K. Wang , Y. Zhou , A. Shamim , L.u Zheng , X. Wang , W. Huang , Adv. Mater. 2024, 36, 2400333. 10.1002/adma.202400333 38652082

[94] J. Kim , A. Banks , Z. Xie , S. Y. Heo , P. Gutruf , J. W. Lee , S. Xu , K.‐I.n Jang , F. Liu , G. Brown , J. Choi , J. H. Kim , X. Feng , Y. Huang , U. Paik , J. A. Rogers , Adv. Funct. Mater. 2015, 25, 4761.

[95] J. Kim , P. Gutruf , A. M. Chiarelli , S. Y. Heo , K. Cho , Z. Xie , A. Banks , S. Han , K.‐I. n Jang , J. W. Lee , K. T. Lee , X. Feng , Y. Huang , M. Fabiani , G. Gratton , U. Paik , J. A. Rogers , Adv. Funct. Mater. 2017, 27, 1604373. 28798658 10.1002/adfm.201604373 PMC5545889

[96] O. D. Incel , Comput. Networks 2011, 55, 3081.

[97] B. Yao , H. Yang , Sci. Rep. 2016, 6, 39012. 27966576 10.1038/srep39012 PMC5155286

[98] P. C. Franzone , L. F. Pavarino , A. Tosin , Modeling, Simulation and Applications 2014, 13, 175.

[99] K. W. Chen , L. Bear , C. W. Lin , Sensors 2022, 2331. 35336502 10.3390/s22062331 PMC8951148

[100] B. Taccardi , B. B. Punske , R. L. Lux , R. S. Macleod , P. R. Ershler , T. J. Dustman , Y. Vyhmeister , J Cardiovasc Electrophysiol 1998, 9, 773. 9684726 10.1111/j.1540-8167.1998.tb00965.x

[101] H. S. Oster , B. Taccardi , R. L. Lux , P. R. Ershler , Y. Rudy , Circulation 1997, 96, 1012. 9264513 10.1161/01.cir.96.3.1012

[102] A. Intini , R. N. Goldstein , P. Jia , C. Ramanathan , K. Ryu , B. Giannattasio , R. Gilkeson , B. S. Stambler , P. Brugada , W. G. Stevenson , Heart Rhythm 2005, 2, 1250. 16253916 10.1016/j.hrthm.2005.08.019 PMC2000800

[103] Y. Rudy , Int. J. Cardiol. 2017, 237, 13. 28258845 10.1016/j.ijcard.2017.02.104 PMC5441950

[104] B. J. Messinger‐Rapport , Y. Rudy , Math. Biosci. 1988, 89, 79. 10.1016/0025-5564(89)90044-8 2520207

[105] W. W. Good , B. Erem , J. Coll‐Font , B. Zenger , B. M. Horacek , D. H. Brooks , R. S. Macleod , Comput Cardiol 2010, 45, 2018. 10.22489/CinC.2018.351 PMC6648695 31338374

[106] S. B. Kotsiantis , I. D. Zaharakis , P. E. Pintelas , Artif Intell Rev 2006, 26, 159.

[107] R. Caruana , A. Niculescu‐Mizil , ACM International Conference Proceeding Series 2006, 148, 161.

[108] C. M. Eckhardt , S. J. Madjarova , R. J. Williams , M. Ollivier , J. Karlsson , A. Pareek , B. U. Nwachukwu , Knee Surgery, Sports Traumatology, Arthroscopy 2023, 31, 376. 10.1007/s00167-022-07233-7 36378293

[109] A. Coronato , M. Naeem , G. De Pietro , G. Paragliola , Artif Intell Med 2020, 109, 101964. 34756216 10.1016/j.artmed.2020.101964

[110] K. D. Roe , V. Jawa , X. Zhang , C. G. Chute , J. A. Epstein , J. Matelsky , I. Shpitser , C. O. Taylor , PLoS One 2020, 15, e0231300. 32324754 10.1371/journal.pone.0231300 PMC7179831

[111] G. D. Goh , J. M. Lee , G. L. Goh , X. Huang , S. Lee , W. Y. Yeong , Tissue Eng Part A 2023, 29, 20. 36047505 10.1089/ten.TEA.2022.0119

[112] C. H. Lee , H. J. Yoon , Kidney Res Clin Pract 2017, 36, 3. 28904873 10.23876/j.krcp.2017.36.3.224 PMC5592889

[113] K. Tucker , J. Branson , M. Dilleen , S. Hollis , P. Loughlin , M. J. Nixon , Z. Williams , BMC Med. Res. Methodol. 2016, 16, 5. 27410040 10.1186/s12874-016-0169-4 PMC4943495

[114] C. Thapa , S. Camtepe , Comput. Biol. Med. 2021, 129, 104130. 33271399 10.1016/j.compbiomed.2020.104130

[115] J. Ficek , W. Wang , H. Chen , G. Dagne , E. Daley , J. Amer. Medical Informatics Association 2021, 28, 2269. 10.1093/jamia/ocab135 PMC8449619 34333623

[116] B. Pfitzner , N. Steckhan , B. Arnrich , ACM Transactions on Internet Technology (TOIT) 2021, 21, 1.

[117] S. Carpov , T. H. Nguyen , R. Sirdey , G. Constantino , F. Martinelli , in IEEE 9th Int. Conf. on Cloud Computing (CLOUD), IEEE, San Francisco, CA, USA 2017, pp. 593–599.

[118] J. Scheibner , M. Ienca , E. Vayena , BMC Med. Ethics 2022, 23, 121. 36451210 10.1186/s12910-022-00852-2 PMC9713155

[119] J. Bergquist , L. Rupp , B. Zenger , J. Brundage , A. Busatto , R. S. MacLeod , Hearts 2021, 2, 514. 35665072 10.3390/hearts2040040 PMC9164986

[120] D. Jin , E. Sergeeva , W. H. Weng , G. Chauhan , P. Szolovits , WIREs Mechanisms of Disease 2022, 14, e1548. 35037736 10.1002/wsbm.1548

[121] D. A. Kaji , J. R. Zech , J. S. Kim , S. K. Cho , N. S. Dangayach , A. B. Costa , E. K. Oermann , PLoS One 2019, 14, e0211057. 30759094 10.1371/journal.pone.0211057 PMC6373907

[122] M. Graziani , V. Andrearczyk , M. S. M. , H. Müller , Comput. Biol. Med. 2020, 123, 103865. 32658785 10.1016/j.compbiomed.2020.103865

[123] N. Arun , N. Gaw , P. Singh , K. Chang , M. Aggarwal , B. Chen , K. Hoebel , S. Gupta , J. Patel , M. Gidwani , et al., Radiol Artif Intell 2021, 3, 2. 10.1148/ryai.2021200267 PMC8637231 34870212

[124] M. S. Ayhan , L. B. Kümmerle , L. Kühlewein , W. Inhoffen , G. Aliyeva , F. Ziemssen , P. Berens , Med Image Anal 2022, 77, 102364. 35101727 10.1016/j.media.2022.102364

[125] X. Li , M. Li , P. Yan , G. Li , Y. Jiang , H. Luo , S. Yin , International J. Network Dynamics and Intelligence 2023, 2, 93.

[126] L. F. Nakayama , W. G. Mitchell , L. Z. Ribeiro , R. G. Dychiao , W. Phanphruk , L. A. Celi , K. Kalua , A. P. D. Santiago , C. V. S. Regatieri , N. S. B. Moraes , BMJ Open Ophthalmol 2023, 8, 1216. 10.1136/bmjophth-2022-001216 PMC10414056 37558406

[127] C. Sendra‐Balcells , V. M. Campello , J. Torrents‐Barrena , Y. A. Ahmed , M. Elattar , B. Ohene‐Botwe , P. Nyangulu , W. Stones , M. Ammar , L. N. Benamer , et al., Sci. Rep. 2023, 13, 2728. 36792642 10.1038/s41598-023-29490-3 PMC9932015

[128] S. Ali , N. Ghatwary , D. Jha , E. Isik‐Polat , G. Polat , C. Yang , W. Li , A. Galdran , M. Á. G. Ballester , V. Thambawita , et al., Sci. Rep. 2024, 14, 2032. 38263232 10.1038/s41598-024-52063-x PMC10805888

[129] T. Liu , W. Fan , C. Wu , Artif Intell Med 2019, 101, 101723. 31813482 10.1016/j.artmed.2019.101723

[130] M. Chieregato , F. Frangiamore , M. Morassi , C. Baresi , S. Nici , C. Bassetti , C. Bnà , M. Galelli , Sci. Rep. 2022, 12, 4329. 35288579 10.1038/s41598-022-07890-1 PMC8919158

[131] A. Maier , H. Köstler , M. Heisig , P. Krauss , S. H. Yang , Progress in Biomedical Engineering 2022, 4, 022002.

[132] B. Saravi , F. Hassel , S. Ülkümen , A. Zink , V. Shavlokhova , S. Couillard‐Despres , M. Boeker , P. Obid , G. M. Lang , J. Personalized Med. 2022, 12, 509. 10.3390/jpm12040509 PMC9029065 35455625

[133] H. Sahu , R. Kashyap , B. K. Dewangan , 2022 OPJU International Technology Conference on Emerging Technologies for Sustainable Development, Raigarh, Chhattisgarh, India, February, 2023.

